# ‘Dementia risk reduction in the African context: Multi‐national implementation of multimodal strategies to promote healthy brain aging in Africa (the Africa‐FINGERS project)

**DOI:** 10.1002/alz70860_099596

**Published:** 2025-12-23

**Authors:** Chinedu T Udeh‐Momoh, Miia Kivipelto, The Africa‐FINGERS Program Investigators

**Affiliations:** ^1^ Aga Khan University, Nairobi, Nairobi, Kenya; ^2^ Wake Forest University, School of Medicine, Winston‐Salem, NC, USA; ^3^ FINGERS BRAIN HEALTH INSTITUTE, Solna, Stockholm, Sweden; ^4^ The Ageing Epidemiology (AGE) Research Unit, School of Public Health, Imperial College London, London, United Kingdom; ^5^ Division of Clinical Geriatrics, Center for Alzheimer Research, Department of Neurobiology, Care Sciences and Society, Karolinska Institutet, Stockholm, Sweden; ^6^ FINGERS Brain Health Institute, Stockholm, Sweden

## Abstract

**Background:**

Dementia is a growing global health crisis, with over 150 million cases projected by 2050, disproportionately affecting low‐ and middle‐income countries (LMICs), particularly Africa. Despite evidence that up to 40% of dementia cases could be prevented by addressing modifiable risk factors, there remains a critical lack of culturally tailored, evidence‐based dementia prevention programs in Africa. The AFRICA‐FINGERS program aims to bridge this gap by implementing a multimodal, precision prevention strategy, leveraging dementia risk scores and biomarker‐driven detection to identify individuals at high risk. This initiative, embedded within the World‐Wide FINGERS (WW‐FINGERS) network, pioneers dementia risk reduction by integrating community‐based system dynamics (CBSD) approaches and deep cultural phenotyping to tailor interventions to the African context.

**Methods:**

AFRICA‐FINGERS is a multi‐country collaboration designed to co‐develop and implement culturally sensitive, multidomain interventions targeting dementia risk factors. The study harmonizes methodologies with the landmark FINGER trial while incorporating contextual determinants identified through ethnographic research, including multidimensional poverty, social connectedness, and nutritional disparities. The project will: * Recruit at‐risk older adults using validated dementia risk scores, supported by vanguard cohorts (e.g., AD‐Detect Kenya). * Conduct biomarker‐informed stratification, integrating blood‐based ADRD biomarker analysis and deep immunophenotyping. * Implement community‐driven interventions such as financial literacy programs, mindfulness‐based cognitive training, and culturally adapted lifestyle modifications. * Facilitate cross‐cohort comparisons between Africa‐FINGERS and US‐POINTER, informing risk reduction strategies for Black Africans in Africa and the diaspora. * Leverage international stakeholder collaboration (e.g., WW‐FINGERS, Global Brain Health Institute, Davos Alzheimer's Collaborative) to build equitable frameworks for research capacity strengthening.

**Results:**

Preliminary findings indicate that common Western‐derived dementia risk scores require contextual adaptation for African populations. Key risk factors identified include cardiometabolic disorders, malnutrition, social isolation, and underdiagnosed vascular conditions, which impact cognitive aging. Ongoing validation of multi‐biomarker profiling to enhance risk stratification and intervention personalization will be presented.

**Conclusions:**

AFRICA‐FINGERS is the first coordinated effort to implement multimodal dementia prevention in Africa, integrating precision prevention and culturally adapted interventions. Through harmonizing strategies with global dementia prevention efforts, this initiative will advance brain health equity, strengthen research capacity, and inform scalable policy frameworks for dementia risk reduction in Africa and globally.

